# Moderation-Mediation Effects in Bilingualism and Cognitive Reserve

**DOI:** 10.3389/fpsyg.2020.572555

**Published:** 2020-09-30

**Authors:** Roberto R. Heredia, Angélique M. Blackburn, Luis A. Vega

**Affiliations:** ^1^ Department of Psychology and Communication, Texas A&M International University, Laredo, TX, United States; ^2^ Department of Psychology, California State University-Bakersfield, Bakersfield, CA, United States

**Keywords:** bilingual cognitive reserve, bilingual moderators, bilingual mediators, cognitive reserve, mediating effects, moderating effects

## Abstract

We first provide a critical review of the existing findings on bilingualism as a contributor to cognitive reserve from moderator-mediator warranting cause-effect research conclusions. We next address the question of direct or indirect effects between bilingualism and neurocognitive protective factors influencing the associated age-related mental deficits. The existing findings support bilingualism as a predictor and as a moderator. Third, we propose cognitive reserve models of bilingualism describing analytical approaches that allow testing of these models and hypotheses related to path strength and causal relationships between predictors, moderators, and mediators. Lastly and most importantly, we suggest using large datasets available *via* open repositories. This can aid in the testing of theoretical models, clarifying the roles of moderators and mediators, and assessing the research viability of multi-causal paths that can influence cognitive reserve. Creating collaborative datasets to test these models would greatly advance our field and identify critical variables in the study of the bilingual aging brain.

## Introduction

This paper is an attempt to clarify some issues that we see interchangeably used in the literature. These issues include mediators and moderators, each of which serves different purposes and has different assumptions and requirements that we clarify. A second purpose is to parse factors that are critical in the study of bilingual aging, specify the extent to which those factors *amplify* or *diminish* an effect (i.e., moderators), and determine whether a particular predictor (e.g., bilingualism) has an indirect effect on the outcome- or dependent-variable that is caused or *mediated* by a third variable. Identifying extraneous variables that moderate or mediate a causal relationship can clarify and advance our field. Third, we suggest other possible analytical approaches to data analyses that might be informative and allow for testing of models and specific hypotheses related to the path strength(s) between and among causal factors. Most importantly, we make a call for using large datasets related to this objective to help us increase understanding of bilingual cognitive research on aging using the Open Science Framework^1^ repositories. This can aid in the testing of theoretical models, clarifying the roles of moderators, and assessing the research viability of multi-causal paths that can influence cognitive reserve. Some data are already accessible through the open access philosophy of PLOS One, especially from those studies involving neurophysiological markers (fMRI and MRI) and others that are not readily accessible but might be in the future through Psychology’s Study Preregistration run by The Center for Open Science.^2^ Creating collaborative datasets would greatly advance our field and identify critical variables in the study of the bilingual aging brain. We first review the general findings regarding the impact of bilingualism on cognitive reserve; second, we review how these important variables have been used, misused, or misinterpreted. Finally, we suggest plausible models that can be used as prototypes in model testing of theories of bilingualism and its influence/relationship to cognitive reserve, through experimental or non-experimental data.

## Cognitive Reserve

As we age, neural networks begin to deteriorate and cognitive abilities decline. In the case of pathologies that impact a large percentage of elderly adults, such as Alzheimer’s Disease (AD) and other forms of dementia, this decline has specific trajectories and consequences. However, some individuals experience a delay in exhibiting the initial symptoms of neural pathology and a slowed rate of its progression. Although their brains are already in early stages of deterioration, their cognitive abilities remain protected for a number of years. Socially, mentally, and physically engaging activities appear to mitigate the effects of cognitive decline (Fratiglioni et al., 2004). Of particular interest, mentally engaging activities such as formal education, mentally challenging occupations, and mentally stimulating leisure activities have been associated with resistance to the effects of neuropathology, in terms of expressing symptoms more slowly (e.g., Valenzuela and Sachdev, 2006a,b).

The association of cognitive resilience in the face of pathology and a history of mentally engaging activity has led to the *cognitive reserve hypothesis* (Valenzuela and Sachdev, 2006a,b) posing that *complex mental* (neural) *activity throughout the lifetime protects against dementia by delaying the onset and rate of cognitive decline* (Bialystok et al., 2007, p. 459; but see Van Dijk et al., 2008). Cognitive reserve is related to functional compensation for deterioration of neural pathways that may occur because some people perform cognitive tasks differently as a result of their experience. However, Woumans et al. (2015) point out that this compensation likely occurs through structural changes in the brain as a result of experience. These changes might include a combination of structural and functional factors such as increased resting phosphocreatine levels (Valenzuela and Sachdev, 2006b), better vascular support, enhanced neural conductivity, or compensation for deterioration in one pathway by using other neural pathways (Fratiglioni et al., 2004). Connections between neurons within a network can become strengthened as that network is repeatedly engaged, which modifies the proteins and enzymes along neural synapses. Neural connectivity can increase (or decrease) as the number of postsynaptic dopamine receptors in the mesocorticolimbic system change, neurotransmitter production is modified, synapses become enlarged, and/or dendritic spines grow on the postsynaptic neuron to increase the contribution of incoming information (Bliss et al., 2003; Nithianantharajah and Hannan, 2011; Freret et al., 2015; Blackburn, 2019; Iaccarino et al., 2020). Another possibility is that by engaging in tasks that require a high degree of cognitive effort, neural pathways for performing specific cognitive tasks are reorganized in a way that is more efficient and demands less resources (Bialystok et al., 2007; Woumans et al., 2015). More efficient networks may aid in retention of cognitive abilities when the networks first begin to deteriorate with age or pathology.

Factors associated with building cognitive reserve include social, mental, and physical engagements (Fratiglioni et al., 2004). However, many lifestyle habits associated with cognitive reserve involve frequent cognitive effort or mental exercise. Mentally demanding leisure activities, playing or listening to music, and playing video games have each been linked with increased reserve (e.g., Scarmeas et al., 2001; Bialystok, 2006; Basak et al., 2008). Formal education and occupational status are two of the most frequently identified predictors of preserving cognitive ability (e.g., Stern et al., 1994; Valenzuela and Sachdev, 2006b); however, some studies have shown that higher levels of education are linked to faster progression of AD (Scarmeas et al., 2006), and more demanding occupations are linked to earlier onset of AD symptoms (Woumans et al., 2015). It has been proposed that these factors may build cognitive reserve, but are associated with other risk factors for AD, such as stress (Schweizer et al., 2012). Another explanation is that although high levels of cognitive reserve are linked to delayed onset of AD, in some cases, decline is more rapid once the symptoms manifest (Stern et al., 1995, 1999). Finally, bilingualism, or the ability to speak two or more languages, is a skill that requires cognitive effort and interaction by many individuals on a regular basis. Similar to other mentally stimulating activities, bilingualism is linked to a delay in the onset and diagnosis of dementia (Bialystok et al., 2007), a robust finding that has been replicated multiple times (Anderson et al., 2020; Brini et al., 2020; Paulavicius et al., 2020). Although research on the relationship between the two is nascent, substantial and significant evidence has been found for bilingualism as a crucial factor that increases cognitive reserve.

## Bilingual Cognitive Reserve

Because *bilingual advantage*(s) later in life appear selective to the way in which bilinguals have used their languages, we can speculate that this added value is in fact a *set of advantages* that result from specific language skills that have been engaged across the lifespan. The bilingual advantage effect is the finding that bilinguals outperform monolinguals on tasks that require cognitive control – the ability to reactively and proactively respond to the demands of the environment (Paap et al., 2014). The cognitive advantages are rarely observed in young adults (for reviews and a meta-analysis, see Hilchey and Klein, 2011; Blackburn, 2017, 2018a, respectively), but are more often observed in older bilinguals. Even though behavioral advantages are not always found in young bilinguals, neural activity during cognitive control tasks is sometimes altered in young bilinguals compared to monolinguals (Kousaie and Phillips, 2012; Blackburn, 2013, 2018b). These neural effects in young adults are linked to enhanced behavioral performance in older adults (see also Morales et al., 2015; Kousaie and Phillips, 2017). Thus, as a result of managing their two or multiple languages throughout the lifespan, bilinguals develop *cognitive reserve*. One of the predominant explanations is that when a bilingual utters a word, the translation equivalent is thought to compete for selection, and interference from the competing language must be suppressed so that the appropriate word can be produced. The cognitive effort necessary to inhibit interference in order to remain in one language can develop neural networks involved in *interference suppression*, making this skill more efficient and resilient as the brain degrades with cognitive aging or pathology (Green, 1998; Bialystok et al., 2005, 2009).

The most convincing evidence of bilingual cognitive reserve comes from studies showing that bilinguals are slower to exhibit signs of dementia. This evidence was first reported by Bialystok et al. (2007) and was based on hospital records from dementia patients. Controlling for such variables as gender, education, and employment status, on average, bilinguals showed initial signs of dementia 4 years later than monolinguals, and they were diagnosed 3.2 years later (75.4 years of age for monolinguals vs. 78.6 years for bilinguals). A similar delay in the report of initial symptoms of 5.1 years for bilinguals was observed in a second study by the same research group of individuals diagnosed with AD (Craik et al., 2010).

Since these initial studies, researchers have replicated the results, finding delays in the onset and diagnosis of AD, and other forms of dementia, even when controlling for potential confounds (for a review, see Freedman et al., 2014). Woumans et al. (2015) controlled for education, profession, and socioeconomic status (SES), and reported delays of 4.6 and 4.8 years for onset and diagnosis of AD, respectively. Bialystok et al. (2014) controlled for diet, smoking, alcohol consumption, and physical and social activities. They found a 7.2 year-delay in the onset of AD and 3.5 year-delay in the onset of mild cognitive impairment (but no difference in the progression of the disease over the course of 1 year). These studies show consistent delays related to bilingualism in onset of symptoms and diagnosis of dementia.

Moreover, a more recent meta-analyses including eight studies found that on average, bilinguals exhibit AD symptoms 4.1 years and are diagnosed with AD 2.0 years later than monolinguals (Paulavicius et al., 2020). Another confirmed a 4.7-year delay in AD onset and a 3.3-year delay in the diagnosis of dementia, but no delay in the diagnosis of mild cognitive impairment (Brini et al., 2020). An additional meta-analysis of 21 studies (18 of which provided the age of AD onset and six of which provided AD incidence) found that the delay in manifestation of AD symptoms has a moderate effect size, although the effect size of rate of AD incidence was weaker (Anderson et al., 2020). The overall conclusion from these studies was that the reported effects did not depend on education (Brini et al., 2020), SES, or publication bias (Anderson et al., 2020). Together, these results suggest that bilingualism has a low likelihood of preventing dementia but it is likely to delay dementia by roughly 4 years in manifestation of AD symptoms. Most importantly, the meta-analysis demonstrated the need for high quality studies, which must observe requirements in conceptual-, methodological-, and statistical-rigor, while controlling for third variables such as culture and education (Grundy and Anderson, 2017; Woumans et al., 2017).

The above findings in bilinguals have been corroborated by comparisons of monolinguals and multilinguals showing less degeneration (Liu et al., 2012) and slower progression of dementia in multilinguals (Perani and Abutalebi, 2015). However, it should be noted that while bilinguals and multilinguals are often older than monolinguals at the time of onset and/or diagnosis, dementia has been shown to progress faster in bilinguals (Berkes et al., 2020). This is reminiscent of other factors, such as high levels of education, that are associated with rapid progression of decline after an initial delay of symptom onset (Stern et al., 1995, 1999). Thus, age and education appear to be moderating variables that need to be accounted for in the relationship between bilingualism and cognitive reserve.

## Bilingual and Social Factors

Bilingual studies have controlled for or specifically tested potential factors involved in cognitive reserve. These factors include the number of languages spoken, immigration status, country, age of second language acquisition, and factors known to impact cognitive reserve, such as education, occupational status, SES, premorbid intelligence, stimulating leisure activities, and mental, social, and physical activity (Guzmán-Vélez and Tranel, 2015).

Moreover, individual differences in the degree of bilingualism, such as early age of acquisition (Klein et al., 2014) and using multiple languages (e.g., Kavé et al., 2008; Chertkow et al., 2010; Perquin et al., 2013), appear to increase the likelihood of protection. Greater dementia delays are also sometimes observed for immigrant compared to non-immigrant populations (Chertkow et al., 2010; Mondini et al., 2014). But this effect may be due to the cognitive effort necessary to operate in a non-dominant language, as dominance in a language other than the L1 is associated with higher performance on cognitive tasks (Kavé et al., 2008). Frequently operating in a non-dominant language may also indicate greater proficiency or frequency of use, both of which are potential modulators of the bilingual advantage in cognitive reserve. It is equally possible that simply using two languages regularly enhances cognitive reserve, as dementia delays have been observed in non-immigrant populations regardless of whether the non-dominant (Alladi et al., 2013) or dominant language (Woumans et al., 2015) is spoken most frequently. In fact, the way in which bilinguals use their languages throughout their lives may engage cognitive control networks in different ways and increase cognitive reserve in the form of strengthening specific compensatory networks (for a review, see Blackburn, 2018a). Thus, individual language habits may play a large role in developing cognitive reserve. Importantly, bilingualism appears to interact with other factors known to enhance cognitive reserve, such as education as mentioned previously (cf. Kavé et al., 2008; Gollan et al., 2011; Hack et al., 2019).

## Moderating and Mediating Effects of Bilingualism

So far, we have reviewed the evidence supporting bilingualism as an expression of cognitive reserve (see also, Bialystok et al., 2014; but see Zahodne et al., 2014; Mukadam et al., 2017a,b; Hack et al., 2019; Papageorgiou et al., 2019), in addition to other factors or predictors involved (e.g., education, IQ, occupation, SES, and immigration status). However, relationships between and among (predictors) factors are complex and might be modified by third variables (Fairchild and MacKinnon, 2009). In the experimental research domain, third variables such as confounds are held constant or statistically adjusted – provided these factors are quantitative, or treated as error variance through random assignment or counterbalancing measures. Other third variables include moderators, mediators, and suppressors. Suppressor variables can be identified when (a) the direct and indirect effects on the dependent variable are positive, negative, or vice versa (i.e., reciprocal suppressor); (b) when a predictor is added and receives a negative weight, and all other predictors are positively interrelated and yet the variance accounted for in the dependent variable increases (i.e., negative suppression); and (c) when by itself the predictor may have a very low correlation with the dependent variable but by virtue of a higher correlation with a second predictor, the original predictor’s influence on the dependent variable increases (i.e., zero/classical suppressor).

**Figure 1:**
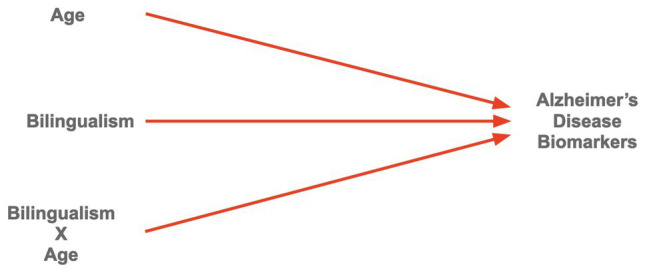
Bilingualism as a moderator interacting with predictor age moderating Alzheimer’s Disease (AD) markers (see Gollan et al., 2011).

Suppressor variables are sometimes called omitted variables (Bollen, 1989, p. 47–54), which are important to considered in structural equation models, where mediator and moderator relationships are examined and where the use of theory requires that all putative causal variables be included. Although we limit our discussion to moderators and mediators (Lancaster, 1999), the presence of omitted/suppressor variables is always lurking, requiring of the same rigor discussed in meta-analysis and requirements of high-quality studies. It is important that researchers have a solid foundation of theory in the bilingual memory field so that they can test the role(s) of suppressor variables that, if omitted or not anticipated, will bias their results. Briefly, a moderator (see Figure 1) qualifies a causal relationship as dependent on another variable in relation to its strength or direction, which we should note are also called *direct effects*. Of note, *modulators* or modulating effects, in the existing literature, are extensively included and are suggestive of moderators or moderating effects. It is not clear, however, if that is the case without delving into the operational definitions of the variables in question and how the analysis is carried out (see Kenny, 2019). Moreover, modulation effects are described even in the absence of interactive effects. A modulator, like a mediator must interact with the other independent variable (IV) or factor to be a *true modulator*. For example, Thorvaldsson et al. (2017) found that IQ moderated terminal decline in old age. Specifically, higher IQs were associated with a delay in onset of terminal decline of up to 1.87 years on speed and 1.96 years on verbal ability. Additionally, Abutalebi et al. (2015) found that age moderated decrease of gray matter volume in the right dorsolateral prefrontal cortex for both bilinguals and monolinguals. An increase in age led to a decrease of gray matter. In the experimental literature, a moderation effect is typically an interactive effect, in which for example, results of an experimental treatment are qualified by gender, age, education, or any other theoretically related variable. In this way, the experimental manipulation is only effective for any variable that further limits, or clarifies, the causal relationship.

**Figure 2:**
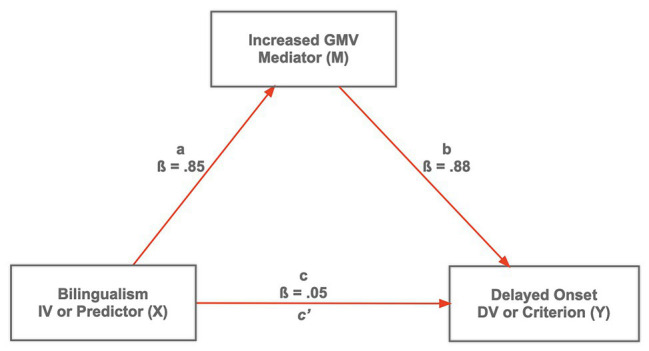
Bilingualism (X) as a predictor variable, and Increased Gray Matter Volume (GMV) (M) mediating Delayed Onset of AD in bilinguals.

Mediators, or intervening effects (see Figure 2), in their simplest expression occur between the predictor and dependent variable in a causal fashion. Mediators account for the relationship between the predictor and dependent variable; the relationship between the predictor and dependent variable is not direct, but indirect *via* a third variable (Baron and Kenny, 1986; Fairchild and MacKinnon, 2009; Petrocelli et al., 2013; Kenny, 2019). Often mediators are hidden, or unknown, only to be identified through theory or previous work in the area. In the classic example, of whether smoking or vaping causes lung cancer, the mediating variable (which could be empirically measured) would be tissue damage. That is, the causal effect between smoking and lung cancer would be an indirect one. We should note that identifying or predicting moderators and mediators in bilingualism research has not been a standard practice. For example, in a search of 69 recent articles consulted for this paper, we found that mediating effects have been of little focus (*n* = 6), with greater emphasis on moderating (*n* = 8) or modulating (*n* = 18) effects, and with approximately 50% (35/69) not giving any consideration to such effects.

### Moderator

Bilingual research in this domain seeks to identify which variables (e.g., SES, IQ, education, and age) best predict cognitive outcomes associated with the aging brain. At issue is whether bilingualism (or multilingualism), controlling for such possible confounds as SES, IQ, and immigration status, protects against mental decline associated with old age. Regardless of the methodological approaches (experimental vs. correlational), the question of bilingualism and its protective cognitive qualities to aging could be posed in terms of *causality* or predictor-based analytical view assessing its contribution to the DV relative to other competing predictors or IVs. Of note, our operating assumptions are that methodological approaches (i.e., experimental vs. correlational), and analytical statistical techniques are orthogonal; binary responses (correct = 1 and incorrect = 0) that were typically converted into percentages and analyzed employing ANOVA can be best analyzed using generalized linear models or generalized mixed linear models (Jaeger, 2008). We first discuss bilingualism and putative causality in relation to moderators. This is important because moderators create dependency of the IV on the DV that needs to clarify if the causal relationship among the variables is confounded, spurious, or attenuated. We then review statistical paradigms that can best capture model testing in determining how bilingualism can be trusted as a strong predictor of cognitive reserve.

Is there a direct effect between bilingualism and mental faculties in the elderly? That is, what evidence exists or can be predicted of bilingualism as a moderator (see Figure 1) and/or a mediator (See Figure 2)? These questions need to be posed in framing hypothesis and *a priori* predictions in a research project if we expect internally valid results that can be generalizable across time, place, and people.

Figure 1 is an example of bilingualism as a moderator, interacting with the predictor age moderating levels of cerebral spinal fluid (CSF), which are AD biomarkers (Estanga et al., 2017). This interaction, as reported by Estanga et al. (2017) revealed that early bilinguals exhibited lower CSF levels as compared to late bilinguals and monolinguals, especially at older ages. Among other interesting effects moderated by bilingualism were lower prevalence of preclinical AD for early bilinguals. Although other studies discriminate between different levels of bilingualism and number of languages spoken, (e.g., Bialystok et al., 2007; Kavé et al., 2008; Alladi et al., 2013; Perquin et al., 2013; Bak et al., 2014; Zahodne et al., 2014; Woumans et al., 2015; Reyes et al., 2018; Hack et al., 2019), the study of Estanga et al. (2017) is unique in the sense that it is one of the few demonstrations that systematically conceptualizes bilingualism as a moderator, testing it, and clarifying that bilingualism influences CSF levels, which can have an effect on the aging brain. Gollan et al. (2011) provide an additional demonstration of education as a moderator, whereby years of education interacted with bilingualism, as measured by the Boston Naming Test, moderating age of Azheimer’s Disease diagnosis; this moderating effect was driven by the bilinguals with low education. Other published studies also hint to the plausibility of bilingualism as a moderator. However, conclusions to the moderating effects of bilingualism are limited and inconclusive given methodological oversights considering the specific effects of bilingualism as a qualitative (i.e., levels or types), and quantitative (Gollan et al., 2011) and composite predictor variable (Calabria et al., 2020).

To illustrate, consider the above explanation and description of Figure 1, but including only bilinguals and monolinguals; the bilingual type (early vs. late) would not be central to the hypothesis at hand. Most likely, the interaction between age and bilingualism would show bilinguals with lower CSF levels than monolinguals, especially at advanced age levels (60 years and above). One possible interpretation, and a prevalent one by some scholars, of a significant interaction might be that, lower CSF AD biomarker levels are *moderated* by bilingualism, as we make the additional assumption that learning other language(s) has been a long learning experience. However, it is important to note that language group (bilingual vs. monolingual or single vs. dual language), in this particular case, would be a plausible moderator but not bilingualism, though the intuition would be probably in the right direction (see Thorvaldsson et al., 2017; Kraemer et al., 2019, for excellent examples of moderation analyses). One possible objection to our argument is that levels of *bilingualism* as a factor could operationally be defined as *bilingual* vs. *nonbilingual*, where the monolingual group is actually a level of the variable. [It would be correct, however, if the focal factor’s levels involve number of languages spoken (e.g., monolingual, bilingual, trilingual, etc.)]. This would be, without any doubt, a classic case of construct validity, and one would have to rely on the extensive literature on bilingualism and the various ways in which bilingualism can be classified or conceptualized (e.g., simultaneous, early, late, compound, coordinate, sequential, successive, low, high proficiency, beginner, and advanced; Heredia and Cieślicka, 2014; see also Gollan et al., 2011; Calabria et al., 2020). Clearly, this research domain would benefit from studies specifically looking at the moderating effects of bilingualism on the aging brain. Excellent literature reviews of the effects of bilingualism and cognitive reserve are provided by Calvo et al. (2016) and Klimova et al. (2017), and recent meta-analyses by Mukadam et al. (2017b); Anderson et al. (2020); Brini et al. (2020); Paulavicius et al. (2020). None of these articles make reference to the plausibility of bilingualism as a moderator, or equally if not more important, as a mediator, to which we now turn.

### Mediator

What is the relationship between bilingualism and neuro-cognitive mental decline associated with old age? Is there a direct (causal) relationship between bilingualism and neurocognitive protective effects such as diet, education, intelligence, or other internal agents that in turn influence (mediating causal effects) associated with the age-related mental deficits of the aging bilingual? A review of the existing bilingual aging literature point to an indirect effect as summarized by Figure 2 [e.g., Luk et al., 2011; Schweizer et al., 2012; Perquin et al., 2013; Abutalebi et al., 2014, 2015; Perani et al., 2017; Reyes et al., 2018; cf. Rossi et al., 2017; cf. Kavé et al., 2008; Mukadam et al., 2017a,b; and see Grady et al., 2015, for a similar proposed mediation model with *lifelong use of two languages* as predictor, and *stronger (neuro) functional connections* and *greater (neuro) modulation of task-related activity* mediating executive control].

Figure 2 describes a mediation model with two causal paths: The direct link, *path c*, between Bilingualism as predictor (X) and Delayed Onset of Alzeimer’s Disease as criterion (Y); direct link, *path b*, between Increased GMV (M) and Y. There is also *path a*, the possibility to which most of the evidence summarized points (see Grady et al., 2015, for a similar argument). Figure 2 provides a simple and straightforward testable model; it would be possible, indeed, to include possible moderators (e.g., education and SES) and mediators (e.g., executive control; see Petrocelli et al., 2013, for a discussion of complex moderation-mediation models; cf. Grady et al., 2015). Whether bilingualism has a direct (*path c*) or indirect effect (*paths a* + *b*) on Delayed Onset of Alzheimer’s or Mental Agility remains to be empirically verified. Utilizing the appropriate regression-based analytical techniques (e.g., path analysis or structural equation modeling), the hypotheses is that *path a* and *b* would reveal large unstandardized weights (*B*s) or standardized regression coefficients (*ßs*; see Figure 2), as compared to a nonsignificant *Path c'* after controlling for *M* could be tested (e.g., Preacher and Hayes, 2008; Fairchild and MacKinnon, 2009; Yzerbyt et al., 2018; see Sauter et al., 2019, for an excellent demonstration of a mediation analysis related to aging and cognitive reserve). Methodological paradigms do not and should not determine the statistical approach. ANOVA has been crucial in experimental psychology; however, other regression-based-statistical techniques (e.g., linear mixed models and generalized mixed models) are crucial in model testing (e.g., Ljungberg et al., 2013; Perquin et al., 2013; Zahodne et al., 2014; Klein et al., 2016; Hack et al., 2019) and overcoming limitations of traditional regression analysis in dealing with repeated measures designs (Lorch and Myers, 1990). Indeed, these statistical techniques required more attention (cf. Bak et al., 2014; Xie and Zhou, 2020).

**Figure 3:**
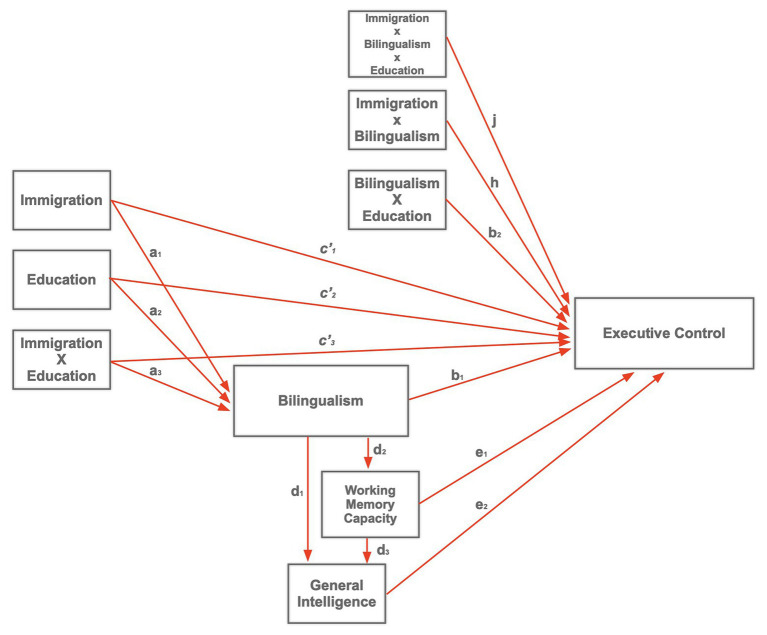
A hypothetical mediation (bilingualism) and moderation (immigration, education, immigration × education; immigration × bilingualism × education, immigration × bilingualism, and bilingualism × education) model of bilingual cognitive reserve (cf. Fairchild and MacKinnon, 2009); working memory capacity (attention span), and general intelligence (cf. Martin et al., 2020).

The existing literature, thus far, supports bilingualism as a predictor and as a moderator. We now consider the viability of bilingualism, as measured in a continuum (Gollan et al., 2011; see also Calabria et al., 2020) as a plausible mediator. Mediators, as argued by Baron and Kenny (1986) *explain how external physical events take on internal psychological significance* (p. 1176). So, a mediator could conceivably be viewed as a mechanism that might explain *why* bilinguals, for example, consistently outperform monolinguals on attentional tasks tapping into executive control (but see Paap, 2018). At a very general level, it might come across as a reasonable explanation. However, from a moderator-mediator perspective, for a mediator status, the causal effects of bilingualism can be predicted from other predictors or markers. Possible predictors of bilingualism or multilingualism can include positive attitudes toward multiculturalism and diversity, language-group/ethnicity as positively viewed, language-group/ethnicity as politically significant, levels of education, SES and immigration status (see for example Simonton, 2008). Additionally, bilingualism could be a conglomeration or a multidimensional composite of multiple predictors and moderators interacting (see for example, Calabria et al., 2020). Figure 3 provides such possibility. The model described in the figure suggests a structure in which immigration as a predictor interacts with education, the moderator, to predict bilingualism as a mediator. In turn, bilingualism develops a causal path (*b_1_*) to the Executive Control, the dependent variable.

If bilingualism is a mediator, a prediction would be that paths *a_3_* and *b_1_* would reveal large and significant *ßs* relative to *a_1_*–*a_2_*, with nonsignificant paths for *c'_1_*–*c'_3_* (see discussion above) and the potential moderating effects of the three-and two-way interactions described by paths *j*, *h*, and *b_2_*. Noticed that the model in Figure 3 could be modified further to incorporate working memory capacity. If we are to assume that bilinguals have larger working memory capacity, a correlate of cognitive reserve, it can be assumed that high working memory capacity in bilinguals moderates executive control. However, Martin et al. (2020) found that working memory capacity (*path e_1_*) was not significant once general intelligence was included into the model. That is, they found no moderation effect, but rather that the role of working memory capacity on the effect of learning (i.e., cognitive reserve) was strictly mediated through intelligence (*paths d_3_ and e_2_*). This is but a small glimpse on the power of using large datasets and including moderating and mediating effects in models of bilingualism and the aging brain. These proposed models, could very well be modified, and improved even further, and tested using newly developed procedures greatly simplified by existing open source statistical program (e.g., med-mod procedure of jamovi.org). To our knowledge, no study, correlational or otherwise, has addressed this possibility employing such statistical techniques as confirmatory factor analysis or structural equation modeling. Existing studies with large datasets (Kavé et al., 2008; Alladi et al., 2013, 2016; Ljungberg et al., 2013; Bak et al., 2014; Zahodne et al., 2014; Mukadam et al., 2017a; Hack et al., 2019) if made accessible *via* the Open Science Network would be a positive step in developing and testing bilingual models of bilingual cognitive reserve.

## Moderation-Mediation Analytical Issues

To systematically identify other possible third variables, moderating or mediating bilingual cognitive reserve effects and the bilingual aging process, additional multivariate studies (i.e., correlational) of the type reported by Kavé et al. (2008); Ljungberg et al. (2013); Bak et al. (2014); Zahodne et al. (2014); Alladi et al. (2016); Klein et al. (2016); Mukadam et al. (2017b); Burke et al. (2019); and Hack et al. (2019) are crucial for model testing and advancing the field. As it has been argued (e.g., Cronbach, 1975), variance of third variables such as moderators and mediators go often unaccounted for. Typically, these factors are not identified, measured, or analyzed appropriately. While it is true that theoretically important variables in an experiment, for example, can be treated as covariates, this can only be true if the factor is a quantitative one (e.g., Gollan et al., 2011). Many third variables relevant to bilingualism may not meet that standard such as monolingual versus bilingual, immigrant versus non-immigrant, or even the ubiquitous variable of gender. The use of hierarchical regression, as in linear mixed modeling approaches, in particular which theoretically considers how variables should be entered into the regression equation, would allow us to estimate variance value added by each variable. With this logic, it can be seen that a moderator is simply the interaction entered into the regression equation, with significance testing to assess for variance added over and beyond the DV or main predictor, in addition to other indexes such as effect size for the interaction (see moderator discussion section). Test of a moderator in regression is also said to be a test of the direct effect of that variable on the dependent variable (*path c* in Figure 2; and all paths in Figure 3 with the exceptions of a_1–3_ and d_1–3_). In turn, a mediator is said to be an indirect effect. As stated earlier, the influence of a predictor is mediated through a theoretically important *third* variable, such that the total effect on the dependent variable is the product of the predictor times the third variable (product of *paths a* and *b* in Figure 2; *paths a*_1–3_ and d_1–3_ in Figure 3). Although our previous discussion placed multiple regression at the discussion of moderators and mediators, the important aspect for the current discussion is that non-experimental approaches can be useful in assessing the effects of third variables.

Furthermore, to better understand the role of moderators (direct effects) and mediators (indirect effects), latent variable models can be constructed and tested. Such approach uses regression as a foundation, but only with multiple structural equations that allow for model testing using best fit indexes, and model comparisons (see discussion above). Again, as in hierarchical regression, theory and *a priori* predictions are required to test these models, which can be said are a reflection of reality, and considers the weight or influence of all variables (e.g., predictors, third variables, exogenous, or endogenous). In a manner similar to confirmatory factor analysis, the models tested can involve the measurement model (observable variables such as education, bilingual type, and learning contextual modes) or the latent, or construct model (unobserved variables such as cognitive reserve). While the computational mathematics behind such models is complex, interested readers are directed to Kenny (2019) for an excellent exposition of these techniques. In a nutshell, structural equation modeling involves regression, factor analysis, and often the maximum likelihood estimation of fitting functions. For instance, a *chi*-square statistic is used to accept (nonsignificant) or reject (significant) the proposed model, in addition to several indexes that assess fit (closer to 1.0, the better). As stated, open source statistical programs (e.g., jamovi.org and jasp-stats.org) have significantly simplified the analytical process without having to learn the complicated computations. With most new statistical programs being menu driven, it is nonetheless critical that researchers planning to use structural equation modeling have basic, practical knowledge of what those statistical tools entail. We recommend Kenny (2019) for an assortment of sources for mastery of these revolutionary statistical techniques.

## Conclusion

Bilingualism is just one of many factors thought to protect against age- and pathology-related cognitive decline. Bilingualism is linked to some advantages in cognitive performance and delays in dementia onset and diagnosis. We have reviewed the literature and suggested other possible analytical approaches to data analyses that might be more informative and which allow for testing of models and specific hypotheses related to the path strength(s) between and among causal factors. Most importantly, we suggest using large datasets using the Open Science Framework (see text footnote 1) repositories. This can aid in the testing of theoretical models, clarifying the roles of mediators and moderators, and assessing the research viability of multi-causal paths that can influence cognitive reserve. One potential avenue for data sharing is Psychology’s Study Preregistration run by The Center for Open Science (see text footnote 2). Creating collaborative datasets would greatly advance our field and identify critical variables in the study of the bilingual, aging brain.

## Author Contributions

RH, AB, and LV contributed equally in the development and final product of the present manuscript. All authors contributed to the article and approved the submitted version.

### Conflict of Interest

The authors declare that the research was conducted in the absence of any commercial or financial relationships that could be construed as a potential conflict of interest.

## Footnotes

^1^https://osf.io/

^2^www.cos.io
